# Associations between urate-lowering therapy and the risk of type 2 diabetes mellitus

**DOI:** 10.1371/journal.pone.0210085

**Published:** 2019-01-07

**Authors:** Hsin-Wen Chang, Ya-Wen Lin, Ming-Hung Lin, Yu-Ching Lan, Ruey-Yun Wang

**Affiliations:** 1 Department of Public Health, China Medical University, Taichung, Taiwan; 2 Center for General Education, Hsuan Chuang University, Hsinchu City, Taiwan; 3 School of Nursing, College of Health Care, China Medical University, Taichung, Taiwan; 4 Department of Pharmacy, Tajen University, Pingtung, Taiwan; 5 Department of Health Risk Management, China Medical University, Taichung, Taiwan; International University of Health and Welfare, School of Medicine, JAPAN

## Abstract

**Background:**

Gout is independently associated with increased risk of type 2 diabetes mellitus (T2DM). Urate-lowering therapy (ULT) might be beneficial in lowering the risks of T2DM. Therefore, we conducted a nested case-control study to evaluate the associations between ULT and T2DM.

**Methods:**

This study retrieved the data of 29,765 gout patients from the period of 1998–2010 by using data from Taiwan’s National Health Insurance Research Database. Controls (n = 59,530) were matched at a 1:2 ratio by age, sex, and region. Multivariate Cox proportional hazards regression were performed to examine the dose-dependent relationship between ULT and T2DM.

**Results:**

The adjusted Hazard ratio (HR) for the association of T2DM with allopurinol or benzbromarone exposure was 1.17 (95% confidence interval (CI) 1.07–1.28) and1.09 (95% CI 1.03–1.15), respectively. The HR for the cumulative allopurinol dose was 0.87 (95% CI 0.71–1.07) for patients with dose ≤1.3 mg/day and was 1.31 (95% CI 1.13–1.52) for those with a dose >15.2 mg/day. Similarly, the HR for the cumulative benzbromarone dose was 0.85(95% CI 0.75–0.96) for patients with a dose ≤1.3 mg/day and 1.42 (95% CI 1.30–1.55) for patients with a dose>9.4 mg/day, respectively. Moreover, the average exposure dose of >100 mg/day for allopurinol and >100 mg/day for benzbromarone was associated with a 1.28-fold (95% CI 1.11–1.48) and 1.47-fold (95% CI 1.23–1.76) T2DM risk respectively. The HR for patients in aged >50 years group with cumulative dose ≤1.3 mg/day of allopurinol or benzbromarone had lower risk of T2DM (HR = 0.74, 95% CI 0.58–0.94 for allopurinol; HR = 0.79, 95% CI 0.69–0.90 for benzbromarone).

**Conclusion:**

Gout patients with prolonged ULT and a high dose of ULT were associated with a significant increase in T2DM risk. Although gout patients with age greater than 50 years and a lower dose of ULT may be beneficial in lowering T2DM risk, further clinical studies need to be confirmed these associations.

## Introduction

The prevalence of diabetes in the WHO report was estimated to be 422 million peoples throughout the world [1]. Uric acid level and hyperuricemia are one of the underlying pathways of gout and a well-studied risk factor for gout [2]. Several studies have investigated that the serum uric acid levels and gout are associated with the metabolic syndrome[3] and have an independent impact on the risk of cardiovascular disease [4, 5] and developing type 2 diabetes mellitus (T2DM)[6, 7]. Randomized trials have reported that lowering uric acid by using allopurinol improves insulin resistance in asymptomatic hyperuricemic individuals [8, 9], and similar improvement in insulin resistance has been observed with the use of benzbromarone as urate-lowering therapy (ULT)[10]. Although ULT might be beneficial in lowering T2DM risk, observational studies have demonstrated that gout may be independently associated with an increased risk of T2DM [11, 12]. The association between ULT and serum uric acid are still controversial in T2DM.

Allopurinol, a xanthine oxidase inhibitor has been approved worldwide to first-line treatment for gout patients with asymptomatic hyperuricemia and comorbid renal or cardiovascular diseases[13]. Xanthine oxidase inhibitors block the synthesis of uric acid and can be used regardless of whether urate is overproduced [14]. Uricosuric drugs including probenecid, sulfinpyrazone, and benzbromarone, which are second-line therapies for gout, block renal tubular urate reabsorption [14, 15]. Uricosuric drugs predominantly act on urate anion exchanger 1—an organic anion transporter—to prevent the reuptake of uric acid at the proximal renal tubule, thus increasing the renal excretion of uric acid[16, 17]. Benzbromarone (not available in the United States) may be prescribed to patients with mild-to-moderate renal insufficiency but is potentially hepatotoxic, whereas probenecid and sulfinpyrazone are generally ineffective in patients with renal impairment[14]. Uricosuric agents are more commonly prescribed than are xanthine oxidase inhibitors[18]. Owing to this study, a higher proportion of gout patients in Taiwan are using benzbromarone (approximately 84.8%, n = 25 254) than allopurinol (57.8%, n = 17 199).

To determine whether long-term or excessive ULT protects against **T2DM** risk, we retrospectively analyzed data from a population-based database of Taiwan and evaluated the relationship between ULT and T2DM risk.

## Material and methods

### Data source and study population

Data analyzed in this study were retrieved from the Taiwan National Health Insurance Research Database (NHIRD), which was established in 1995 and collects all claims of those insured under the National Health Insurance (NHI) program. The program covers more than 98% of the total population (23,000,000) and has contracted with 97% of the hospitals and clinics in Taiwan[19]. For this analysis, we used data from Longitudinal Health Insurance Database 2010 (LHID2010), which is a nationwide database including the claims data of 1 million individuals randomly selected from all insurants in the NHIRD. We conducted a nested case-control study by using data from the NHIRD for the period between January 1998 and December 2010. The diagnoses in the NHIRD are coded according to the International Classification of Diseases, Ninth Revision, Clinical Modification diagnostic (ICD-9-CM). The diagnoses in the claims data of the NHIRD are primarily used for administrative purposes, and anonymity of the data is ensured by assigning identification numbers. The study protocols were reviewed and approved by the China Medical University Ethics Review Committee.

### Inclusion and exclusion criteria

**Fig 1:**
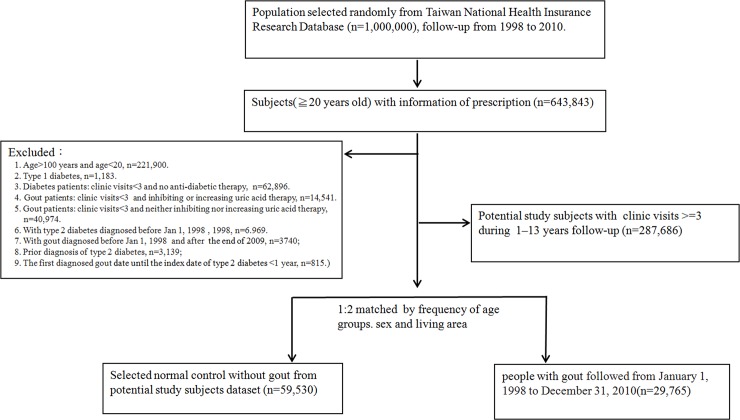
Schematic of the samples selection process for association between urate-lowering therapy and risk of type 2 diabetes mellitus.

The inclusion criteria were patients newly diagnosed with gout and age ≥20 years, and the study population was followed up for 13 years. The exclusion criteria were age >100 years and age <20 years, type 1 diabetes (250.x1 or 250.x3, with x = 0–9), type 2 diabetic patients with clinic visits <3 and no antidiabetic therapy, gout patients with clinic visits <3 and inhibiting or increasing uric acid therapy, gout patients with clinic visits <3 and no inhibiting or increasing uric acid therapy, patients diagnosed with T2DM before December 31, 1998, patients diagnosed with gout before January 1, 1998, and after January 1, 2010, prior diagnosis of T2DM and the first diagnosis date for gout until the index date of T2DM <1 year. The more detailed information was presented in Fig 1.

### Ascertainment of gout

This study comprises 29 765 gout patients and 59 530 controls (matched at a 1:2 ratio). The primary case definition of gout was having a physician-recorded primary diagnosis (ICD-9-CM 274.x) at an outpatient or inpatient visit. To evaluate the robustness of gout case ascertainment, gout was defined as ≥3 clinic visits with ULT. The agents for ULT inhibited uric acid production (allopurinol, Anatomic Therapeutic Chemical code M04AA01), and those for ULT increased uric acid excretion (probenecid, M04AB01; sulfinpyrazone, M04AB02; and benzbromarone, M04AB03).

We calculated the cumulative exposure dose for ULT, which was derived by dividing the total exposure dose for ULT by the total follow-up days (by the first treatment date for gout among gout patients until the index date of T2DM or to the study end with no events). Simultaneously, we calculated the annual exposure day, which was derived by dividing the total exposure days of ULT by the total follow-up time, and the average exposure dose for ULT, which was derived by dividing the total exposure dose for ULT by the total exposure days. We classified the cumulative exposure dose for allopurinol into the following categories according to the quartile method: none, >0–1.3, >1.3–4.2, >4.2–15.2, and >15.2, and that for benzbromarone was categorized as follows: none, >0–1.3, >1.3–3.4, >3.4–9.4, and >9.4. In addition, the quartile method was adopted to classify the annual exposure days for allopurinol (none, >0–2.5, >2.5–8.8, >8.8–37.0, and >37.0) and benzbromarone (none, >0–6.6, >6.6–17.6, >17.6–49.1, and >49.1). We classified the average exposure dose for allopurinol in the following categories: none, >0–100, >100–200, >200–300, and >300, and that for benzbromarone was categorized as follows: none, >0–50, >50–100, and >100.

### T2DM outcome assessment

T2DM events were defined as a new occurrence of T2DM events (ICD-9-CM code 250.x0 or 250.x2, with x = 0–9). T2DM was defined as ≥3 clinic visits and antidiabetic treatment. The oral antidiabetic agents were metformin (A10BA), sulfonylureas (A10BB), meglitinides (A10BX), thiazolidinediones (A10BG), and α-glucosidase inhibitor (A10BF). The insulin injection agents were rapid-acting (A10AB), intermediate-acting (A10AC), long-acting (A10AE), and combination (A10AD).

### Assessment of covariates

From the NHIRD, we collected data on the demographic characteristics of age, sex, and region, as well as the comorbidities of myocardial infarction, congestive heart failure, peripheral vascular disease, cerebrovascular disease, rheumatologic disease, renal disease, and alcohol-related diseases, with diagnoses coded according to ICD-9-CM[20].

### Statistical analyses

Continuous variables are presented as mean (standard deviation) or median (interquartile range), and categorical variables are presented as frequencies (percentages). Continuous and categorical variables were analyzed using a *t*-test or chi-square test for comparisons between gout patients and controls. The dose-response relationship and the annual exposure day for ULT were measured. Moreover, the relationship between diverse exposure doses for ULT and the risk of T2DM was analyzed using the Cox proportional hazards model. Multivariate models were adjusted for the covariates of age group, sex, region, and comorbidities. We also conducted subgroup analyses of gout patients on ULT by sex or age to examine their influence. To assess the robustness of our findings, sensitivity analyses were performed by simultaneously altering the relative risk of the dose-response relationship between ULT doses and the T2DM risk. P values of less than 0.05 were regarded as significant. All statistical analyses were performed using SAS statistical software, version 9.4 (SAS Institute, Cary, NC, USA).

## Results

### Baseline characteristics of study subjects

**We** presented the baseline characteristics of gout patients and controls in the Table **1**. The mean age was 47.3 years among the gout patients. Thus, we adopted age ≤50 years and age >50 years to classify age-specific subgroups for further analysis in this study. **We** listed only allopurinol and benzbromarone exposure and overall exposure to each agent stratified by T2DM and non- T2DM in Table 2.

**Table 1:** Characteristics of subjects with gout and controls.

	Gout	Controls	P value
N	29765	59530	
Type 2 diabetes, n(%)	3940 (13.2)	6334 (10.6)	<0.0001
Oral anti-diabetic therapy (ATC code), n(%)			
Metformin (A10BA)	3540 (11.9)	5753 (9.7)	<0.0001
Sulfonylureas (A10BB)	3222 (10.8)	5675 (9.5)	<0.0001
Meglitinides (A10BX)	599 (2.0)	1173 (2.0)	0.6714
Thiazolidinediones (A10BG)	623 (2.1)	1534 (2.6)	<0.0001
α-glucosidase inhibitor (A10BF)	825 (2.8)	1695 (2.8)	0.5202
Insulin injection therapy (ATC code), n(%)			
Rapid-acting (A10AB),	484 (1.6)	960 (1.6)	0.8807
Intermediate-acting (A10AC)	89 (0.3)	276 (0.5)	0.0003
Combination (A10AD)	61 (0.2)	205 (0.3)	0.0003
Long-acting (A10AE)	60 (0.2)	183 (0.3)	0.0042
Age mean(SD), years	55.4 (16.2)	55.3 (16.1)	0.2865
Gout diagnosis age (SD), years	47.3 (15.9)		
Age group, n(%)			
20 to 30	1542 (5.2)	3158 (5.3)	
>30 to 40	4161 (14.0)	8370 (14.1)	
>40 to 50	5794 (19.5)	11466 (19.3)	
>50 to 60	7187 (24.1)	14459 (24.3)	
>60 to 70	4702 (15.8)	9450 (15.9)	
>70 to 80	4001 (13.4)	7934 (13.3)	
>80	2378 (8.0)	4693 (7.9)	0.9353
Gender, n(%)			
Men	24561 (82.5)	48995 (82.3)	
Women	5204 (17.5)	10535 (17.7)	0.4303
Region, n(%)			
Northern	13788 (46.3)	27669 (46.5)	
Central	7177 (24.1)	13936 (23.4)	
Southern	7686 (25.8)	15909 (26.7)	
Eastern	825 (2.8)	1487 (2.5)	
Offshore islets and other	289 (1.0)	529 (0.9)	0.9423
Comorbidities, n(%)			
Myocardial infarction	377 (1.3)	451 (0.8)	<0.0001
Congestive heart failure	2256 (7.6)	2121 (3.6)	<0.0001
Peripheral vascular disease	1448 (4.9)	1604 (2.7)	<0.0001
Cerebrovascular disease	3580 (12.0)	4982 (8.4)	<0.0001
Rheumatologic disease	1303 (4.4)	982 (1.6)	<0.0001
Renal disease	2162 (7.3)	1285 (2.2)	<0.0001
Alcohol-related diseases	838 (2.8)	598 (1.0)	<0.0001

SD: standard deviation; Comorbidities were defined as ≥3 outpatient claims.

Continuous and categorical variables were analyzed using a *t* test and chi-square test for comparisons between gout patients and controls.

**Table 2:** Only allopurinol or benzbromarone use in gout patients.

		Inhibiting uric acid production M04AA01 (Allopurinol)					Increasing uric acid excretion M04AB03(Benzbromarone)	
	Total	Type 2 diabetes	Non-type 2 diabetes	P	Total	Type 2 diabetes	Non-type 2 diabetes	P
Males, n	3182	375	2807		9787	975	8812	
Follow-up duration, median(IQR), years	6.5 (3.6–9.2)	4.5 (2.5–6.8)	6.7 (3.8–9.4)	<0.0001	6.7 (3.6–9.4)	4.4 (2.5–6.8)	7 (3.9–9.5)	<0.0001
Total clinic visits, median(IQR), frequencies	3 (2–8)	4 (2–11)	3 (1–8)	0.0028	4 (2–10)	5 (2–13)	4 (2–10)	0.0002
Total drug use, median (IQR), days	43 (14–147)	56 (14–180)	42 (14–141)	0.0316	90 (30–224)	97 (30–280)	88.5 (30–219)	0.0683
Total drug tablets, median (IQR), quantities	70 (28–212)	90 (30–294)	65 (28–203)	0.0040	97 (42–252)	115 (42–341)	93 (42–240.5)	0.0070
Total dosage, median (IQR), mg	7650 (2800–22700)	9100 (3000–29900)	7000 (2800–22400)	0.0096	6000 (2800–15600)	7000 (2800–20050)	6000 (2800–15100)	0.0178
Total dosage(mg) /Follow-up duration(years×365 days)	4.2 (1.3–15.1)	7.6 (2.3–24.7)	3.8 (1.3–14)	<0.0001	3.3 (1.2–9.1)	5.2 (1.8–14.7)	3.1 (1.2–8.6)	<0.0001
Total drug use(day)/Follow-up duration(years)	8.6 (2.6–36.3)	14.4 (4.6–56.1)	8.1 (2.3–33.8)	<0.0001	17.2 (6.5–48)	26.8 (8.9–76.7)	16.5 (6.3–45.3)	<0.0001
** Total dosage(mg)/Total drug use(day)**	**200 (100–300)**	**200 (100–300)**	**200 (100–300)**	**0.3930**	**63.7 (50–100)**	**67.6 (50–100)**	**63.5 (50–100)**	**0.0291**
Females, n	911	176	735		2536	507	2029	
Follow-up duration, median(IQR), years	6.5 (3.4–9.2)	3.9 (2.2–6.4)	7.1 (4.1–9.6)	<0.0001	6.5 (3.5–9.1)	4.1 (2.2–6.3)	7.1 (4–9.5)	< .0001
Total clinic visits, median(IQR), frequencies	4 (2–9)	4 (2–14.5)	4 (1–8)	0.0651	4 (2–10)	5 (2–12)	4 (2–9)	0.0648
Total drug use, median (IQR), days	53 (14–168)	52.5 (16.5–233)	53 (14–152)	0.1744	90 (36–235)	91 (35–266)	90 (37–229)	0.6038
Total drug tablets, median (IQR), quantities	79 (28–240)	84 (28–383.5)	78 (24–212)	0.0574	107 (44–261)	116 (42–300)	105 (44–250)	0.2896
Total dosage, median (IQR), mg	8400 (2800–25200)	8700 (2800–38350)	8400 (2700–22400)	0.0762	6250 (2900–16300)	6750 (3000–17200)	6000 (2900–15600)	0.5745
Total dosage(mg) /Follow-up duration(years×365 days)	4.3 (1.2–16.1)	7.5 (1.8–31.8)	3.8 (1.1–13.7)	<0.0001	3.8 (1.3–10.1)	5.6 (2.2–15.1)	3.4 (1.2–9.3)	<0.0001
Total drug use(day)/Follow-up duration(years)	9.3 (2.3–39.3)	13.6 (3.8–87)	8.5 (2.1–34.4)	0.0001	19.7 (7.1–55.1)	29 (11.1–82.2)	17.3 (6.4–47.8)	<0.0001
** Total dosage(mg)/Total drug use(day)**	**183.3 (100–300)**	**200 (100–277.2)**	**180 (100–300)**	**0.9179**	**56.3 (50–100)**	**54.9 (50–100)**	**56.3 (50–100)**	**0.9299**
Combined group, n	4093	551	3542		12323	1482	10841	
Follow-up duration, median(IQR), years	6.5 (3.5–9.2)	4.3 (2.5–6.7)	6.8 (3.8–9.4)	<0.0001	6.7 (3.6–9.3)	4.2 (2.4–6.6)	7 (3.9–9.5)	<0.0001
Total clinic visits, median(IQR), frequencies	3 (2–8)	4 (2–13)	3 (1–8)	0.0004	4 (2–10)	5 (2–12)	4 (2–9)	<0.0001
otal drug use, median (IQR), days	45 (14–150)	56 (15–201)	42 (14–144)	0.0116	90 (32–224)	95 (31–271)	90 (33–222)	0.0314
Total drug tablets, median (IQR), quantities	70 (28–221)	90 (28–329)	69 (28–207)	0.0007	98 (42–254)	116 (42–325)	96 (42–242)	0.0022
Total dosage, median (IQR), mg	7900 (2800–23800)	9000 (3000–33000)	7400 (2800–22400)	0.0021	6000 (2800–15700)	7000 (2800–19000)	6000 (2800–15300)	0.0118
Total dosage(mg) /Follow-up duration(years×365 days)	4.2 (1.3–15.2)	7.6 (2.1–26.8)	3.8 (1.2–13.9)	<0.0001	3.4 (1.3–9.4)	5.4 (1.9–14.9)	3.2 (1.2–8.7)	<0.0001
Total drug use(day)/Follow-up duration(years)	8.8 (2.5–37)	13.8 (4.2–59.2)	8.2 (2.3–33.8)	<0.0001	17.6 (6.6–49.1)	27.7 (9.7–77.7)	16.6 (6.3–45.9)	<0.0001
** Total dosage(mg)/Total drug use(day)**	**200 (100–300)**	**200(100–300)**	**197.9 (100–300)**	**0.5660**	**62.5 (50–100)**	**63 (50–100)**	**62.5 (50–100)**	**0.1631**

IQR: interquartile range.

Continuous variables were analyzed using a *t* test for comparisons between type 2 diabetes and non-type 2 diabetes among gout patients.

### The relative risk for of T2DM with allopurinol or benzbromarone use

As shown in Table 3 and S1 Table, the hazard ratio (HR) for the relationship of T2DM with allopurinol or benzbromarone use was 1.17 (95% CI 1.07–1.28) or 1.09 (95% CI 1.03–1.15), respectively. We found that **T2DM** risk was higher in allopurinol or benzbromarone users than in nonuser controls. Compared with nonuser controls, the HR for the cumulative allopurinol dose was 0.87 (95% CI 0.71–1.07) for patients on ≤1.3 mg/day and were 1.31 (95% CI 1.13–1.52) for those on >15.2 mg/day. Similarly, the HR for the cumulative benzbromarone dose was 0.85 (95% CI 0.75–0.96) for patients on ≤1.3 mg/day and 1.42(95% CI 1.30–1.55) for those on >9.4 mg/day. In sensitivity analysis 1, we similarly showed the dose–response relationship between ULT doses and the T2DM risk. In sensitivity analysis 2, the HR for T2DM increased from 0.92 to 1.45 with allopurinol exposure doses and from 1.06 to 1.47 with benzbromarone exposure doses.

**Table 3:** Association between allopurinol or benzbromarone use and risk of developing type 2 diabetes.

	Type 2 diabetes events, n (%)	Total people, n	Adjusted HR (95% CI)	P value
Allopurinol (M04AA01)				
Cumulative exposure dose*				
Non use	6334 (10.64)	59530	1.00	
Use, mg/day				
>0 to 1.3	89 (8.73)	1019	0.87 (0.71–1.07)	0.1908
>1.3 to 4.2	119 (11.58)	1028	1.12 (0.93–1.34)	0.2293
>4.2 to 15.2	156 (15.26)	1022	1.30 (1.11–1.53)	0.0012
>15.2	187 (18.26)	1024	1.31 (1.13–1.52)	0.0003
** Increasing uric acid excretion**	**1501 (11.94)**	**12566**	**1.09 (1.03–1.15)**	**0.0036**
Combination therapy	1647 (12.57)	13106	1.03 (0.98–1.09)	0.2693
Benzbromarone (M04AB03)				
Cumulative exposure dose				
Non use	6334 (10.64)	59530	1.00	
Use, mg/day				
>0 to 1.3	270 (8.51)	3174	0.85 (0.75–0.96)	0.0092
>1.3 to 3.4	297 (9.84)	3018	0.96 (0.85–1.08)	0.4706
>3.4 to 9.4	374 (12.26)	3050	1.08 (0.97–1.20)	0.1404
>9.4	541 (17.56)	3081	1.42 (1.30–1.55)	<0.0001
** Allopurinol**	**551 (13.46)**	**4093**	**1.17 (1.07–1.28)**	**0.0004**
Probenecid or Sulfinpyrazone	19 (7.82)	243	0.76 (0.48–1.19)	0.2231
Combination therapy	1647 (12.57)	13106	1.03 (0.98–1.09)	0.2594
Sensitivity analysis 1**†**				
Allopurinol (M04AA01)				
Cumulative exposure dose				
Non use	6334 (10.64)	59530	1.00	
Use, mg/day				
>0 to 1.3	379 (9.35)	4055	0.89 (0.80–0.99)	0.0309
>1.3 to 4.2	464 (10.81)	4292	0.99 (0.90–1.09)	0.8522
>4.2 to 15.2	627 (14.05)	4464	1.16 (1.07–1.26)	0.0003
>15.2	728 (16.59)	4388	1.16 (1.07–1.25)	0.0003
Other	1501 (11.94)	12566	1.09 (1.03–1.15)	0.0034
Benzbromarone (M04AB03)				
Cumulative exposure dose				
Non use	6334 (10.64)	59530	1.00	
Use, mg/day				
>0 to 1.3	542 (9.04)	5997	0.86 (0.79–0.94)	0.0006
>1.3 to 3.4	615 (10.3)	5969	0.95 (0.87–1.03)	0.2200
>3.4 to 9.4	828 (12.63)	6558	1.08 (1.00–1.16)	0.0491
>9.4	1128 (16.76)	6730	1.29 (1.21–1.38)	<0.0001
Other	586 (12.99)	4511	1.14 (1.05–1.24)	0.0029
Sensitivity analysis 2‡				
Allopurinol (M04AA01)				
Average exposure dose				
Non use	6334 (10.64)	59530	1.00	
Use, mg/day				
>0 to 100	151 (11.70)	1291	0.92 (0.78–1.08)	0.2927
>100 to 200	193 (15.45)	1249	1.28 (1.11–1.48)	0.0007
>200 to 300	184 (13.28)	1386	1.31 (1.13–1.52)	0.0003
>300	23 (13.77)	167	1.45 (0.96–2.19)	0.0742
** Increasing uric acid excretion**	**1501 (11.94)**	**12566**	**1.09 (1.03–1.15)**	**0.0038**
Combination therapy	1647 (12.57)	13106	1.03 (0.97–1.09)	0.2958
Benzbromarone (M04AB03)				
Average exposure dose				
Non use	6334 (10.64)	59530	1.00	
Use, mg/day				
>0 to 50	601 (11.67)	5148	1.06 (0.97–1.15)	0.1945
>50 to 100	760 (11.92)	6377	1.08 (1.00–1.16)	0.0475
>100	121 (15.16)	798	1.47 (1.23–1.76)	<0.0001
**Allopurinol**	**551 (13.46)**	**4093**	**1.17 (1.07–1.28)**	**0.0005**
Probenecid or Sulfinpyrazone	19 (7.82)	243	0.76 (0.48–1.18)	0.2218
Combination therapy	1647 (12.57)	13106	1.03 (0.98–1.09)	0.2823

Drugs used for increasing uric acid excretion were probenecid (M04AB01), sulfinpyrazone (M04AB02), and benzbromarone (M04AB03)

Combination therapy involved allopurinol and drugs used for increasing uric acid excretion

Adjusted HR was calculated and adjusted for age group, sex, region, and comorbidities by using a Cox proportional hazards regression model.

*Cumulative exposure allopurinol or benzbromarone dose: the accumulated allopurinol or benzbromarone dose divided by the total follow-up days (by the first treat gout date until the index date of type 2 diabetes or to the study end).

**†**Sensitivity analysis 1 was calculated as all allopurinol or benzbromarone use in gout patients (versus controls).

‡Sensitivity analysis 2 was calculated as total dose/total drug use day in gout patients (versus controls).

### Developing risk of T2DM stratified by sex

Table 4 shows the association between allopurinol or benzbromarone use and the **T2DM** risk stratified by sex. The HR for the association of **T2DM** in women, were 1.19 (95% CI 1.02–1.40) for allopurinol use and 1.25 (95% CI 1.13–1.38) for benzbromarone use, respectively. However, only men with allopurinol use had a higher T2DM risk (HR = 1.16, 95% CI 1.05–1.29), and no significantly higher T2DM risk was found among men with benzbromarone use (HR = 1.02, 95% CI 0.95–1.10). Similarly, the higher T2DM risk was observed in patients taking high doses of allopurinol or benzbromarone compared those with nonuser among men and women.

**Table 4:** Association between allopurinol or benzbromarone use and risk of developing type 2 diabetes stratified by sex.

		Men				Women		
	Type 2 diabetes events, n (%)	Total people , n	Adjusted HR (95% CI)	P value	Type 2 diabetes events, n (%)	Total people, n	Adjusted HR (95% CI)	P value
Allopurinol (M04AA01)								
Cumulative exposure dose								
Non use	4693 (9.58)	48995	1.00		1641 (15.58)	10535	1.00	
Use, mg/day								
>0 to 1.3	56 (7.24)	774	0.82 (0.63–1.07)	0.1506	33 (13.47)	245	0.96 (0.68–1.35)	0.7982
>1.3 to 4.2	83 (10.11)	821	1.11 (0.89–1.38)	0.3577	36 (17.39)	207	1.14 (0.82–1.59)	0.4226
>4.2 to 15.2	113 (14.2)	796	1.38 (1.15–1.67)	0.0006	43 (19.03)	226	1.14 (0.84–1.54)	0.3983
>15.2	123 (15.55)	791	1.25 (1.04–1.50)	0.0150	64 (27.47)	233	1.46 (1.14–1.88)	0.0030
Increasing uric acid excretion	985 (9.88)	9972	1.02 (0.95–1.10)	0.5178	516 (19.89)	2594	1.25 (1.13–1.38)	<0.0001
Combination therapy	1293 (11.34)	11407	1.00 (0.94–1.07)	0.9170	354 (20.84)	1699	1.13 (1.01–1.28)	0.0365
Benzbromarone (M04AB03)								
Cumulative exposure dose								
Non use	4693 (9.58)	48995	1.00		1641 (15.58)	10535	1.00	
Use, mg/day								
>0 to 1.3	189 (7.43)	2544	0.84 (0.73–0.97)	0.0207	81 (12.86)	630	0.87 (0.69–1.08)	0.2128
>1.3 to 3.4	198 (8.08)	2450	0.89 (0.77–1.03)	0.1076	99 (17.43)	568	1.13 (0.92–1.39)	0.2315
>3.4 to 9.4	227 (9.49)	2393	0.96 (0.84–1.10)	0.5814	147 (22.37)	657	1.35 (1.14–1.60)	0.0005
>9.4	361 (15.04)	2400	1.37 (1.23–1.52)	<0.0001	180 (26.43)	681	1.55 (1.33–1.82)	<0.0001
Allopurinol	375 (11.79)	3182	1.16 (1.05–1.29)	0.0055	176 (19.32)	911	1.19 (1.02–1.40)	0.0267
Probenecid or Sulfinpyrazone	10 (5.41)	185	0.63 (0.34–1.17)	0.1401	9 (15.52)	58	1.00 (0.52–1.92)	0.9903
Combination therapy	1293 (11.34)	11407	1.00 (0.94–1.07)	0.8957	354 (20.84)	1699	1.14 (1.01–1.28)	0.0356

Drugs used for increasing uric acid excretion were probenecid (M04AB01), sulfinpyrazone (M04AB02), and benzbromarone (M04AB03)

Combination therapy involved allopurinol and drugs used for increasing uric acid excretion

Adjusted HR was calculated and adjusted for age group, region, and comorbidities by using a Cox proportional hazards regression model.

Cumulative exposure allopurinol or benzbromarone dose: the accumulated allopurinol or benzbromarone dose divided by the total follow-up days (by the first treat gout date until the index date of type 2 diabetes or to the study end).

### Developing risk of T2DM stratified by age

**Table 5** shows the association between the allopurinol or benzbromarone use and the type 2 diabetes risk stratified by age ≤50 years and age >50 years. The HR for the association of T2DM with allopurinol or benzbromarone use was 2.28 (95% CI 1.84–2.81) or 1.90 (95% CI 1.64–2.20) in patients aged ≤50 years, respectively. A consistent result was found in those taking high doses. Patients with allopurinol or benzbromarone use by annual exposure day had higher risk for developing T2DM (S2 Table), whereas the HR for patients in aged >50 years group with cumulative dose ≤1.3 mg/day of allopurinol or benzbromarone had lower risk of T2DM (HR = 0.74, 95% CI 0.58–0.94 for allopurinol; HR = 0.79, 95% CI 0.69–0.90 for benzbromarone).

**Table 5:** Association between allopurinol or benzbromarone use and risk of developing type 2 diabetes stratified by age group.

		Age≤50 years				Age>50 years		
	Type 2 diabetes events, n (%)	Total people , n	Adjusted HR (95% CI)	P value	Type 2 diabetes events, n (%)	Total people, n	Adjusted HR (95% CI)	P value
Allopurinol (M04AA01)								
Cumulative exposure dose								
Non use	593 (2.58)	22994	1.00		5741(15.71)	36536	1.00	
Use, mg/day								
>0 to 1.3	22 (4.88)	451	1.82 (1.19–2.79)	0.0058	67 (11.80)	568	0.74 (0.58–0.94)	0.0126
>1.3 to 4.2	27 (6.08)	444	2.31 (1.57–3.40)	<0.0001	92 (15.75)	584	0.98 (0.80–1.20)	0.8460
>4.2 to 15.2	25 (6.25)	400	2.17 (1.45–3.25)	0.0002	131 (21.06)	622	1.20 (1.01–1.43)	0.0375
>15.2	28 (9.24)	303	2.93 (2.00–4.30)	<0.0001	159 (22.05)	721	1.17 (1.00–1.37)	0.0494
Increasing uric acid excretion^¶^	267 (5.19)	5146	1.90 (1.64–2.20)	<0.0001	1234 (16.63)	7420	0.99 (0.93–1.05)	0.7127
Both use*	326 (6.86)	4753	2.31 (2.01–2.65)	<0.0001	1321 (15.81)	8353	0.89 (0.84–0.95)	0.0002
Benzbromarone (M04AB03)								
Cumulative exposure dose								
Non use	593 (2.58)	22994	1.00		5741 (15.71)	36536	1.00	
Use, mg/day								
>0 to 1.3	56 (3.72)	1506	1.45 (1.10–1.90)	0.0083	214 (12.83)	1668	0.79 (0.69–0.90)	0.0006
>1.3 to 3.4	61 (4.59)	1328	1.77 (1.36–2.31)	<0.0001	236 (13.96)	1690	0.86 (0.75–0.97)	0.0191
>3.4 to 9.4	71 (5.96)	1191	2.13 (1.66–2.72)	<0.0001	303 (16.30)	1859	0.96 (0.85–1.07)	0.4415
>9.4	74 (7.38)	1003	2.40 (1.88–3.06)	<0.0001	467 (22.47)	2078	1.29 (1.17–1.42)	<0.0001
Allopurinol	102 (6.38)	1598	2.28 (1.84–2.81)	<0.0001	449 (18.00)	2495	1.05 (0.95–1.15)	0.3684
Probenecid or Sulfinpyrazone	5 (4.24)	118	1.56 (0.65–3.77)	0.3194	14 (11.20)	125	0.66 (0.39–1.12)	0.1257
Both use*	326 (6.86)	4753	2.31 (2.01–2.65)	<0.0001	1321(15.81)	8353	0.89 (0.84–0.95)	0.0003

^¶^Drugs used for increasing uric acid excretion were probenecid (M04AB01), sulfinpyrazone (M04AB02), and benzbromarone (M04AB03)

*Combination therapy involved allopurinol and drugs used for increasing uric acid excretion

Adjusted HR was calculated and adjusted for age group, sex, region, and comorbidities by using a Cox proportional hazards regression model.

Cumulative exposure allopurinol or benzbromarone dose: the accumulated allopurinol or benzbromarone dose divided by the total follow-up days (by the first treat gout date until the index date of type 2 diabetes or to the study end).

## Discussion

The results of this nested case-control study indicated that allopurinol or benzbromarone use is associated with the risk of developing T2DM, particularly in patients receiving high doses of allopurinol or benzbromarone and those with prolonged use. Such consistent result was also found in sex-specific and age-specific subgroup analysis. Compared with gout patients older than 50 years, we found that gout patients younger than 50 years were at a higher risk of T2DM. Uric acid-lowering therapy in gout patients may not be beneficial in lowering T2DM risk, particularly in gout patients younger than 50 years. Although gout patients with age greater than 50 years and a low dose of ULT may be beneficial in lowering T2DM risk, further clinical studies need to be confirmed these associations.

Gout and hyperuricemia have been linked with an increased occurrence of several comorbidities, such as coronary artery disease and hypertension [21], chronic kidney disease [22] or T2DM [23]. However, it is unknown whether the high serum uric acid is a causal factor in the development of these conditions or is a consequence of the manifestation of these disorders. In this study, gout patients older than 50 years with two ULT treatments had a protective effect on T2DM risk (HR = 0.89, 95% CI 0.84–0.95, P = 0.0002). The protective effect was also observed in the low dose of allopurinol or benzbromarone treatment in the older patients group (age >50 years old). The effects of ULT on developing T2DM risk were controversial in the different age groups. Because individuals with gout generally have an increased prevalence of hypertension, a decline in renal function[24] and obesity[25], these co-morbidities are well-known risk factors of T2DM and may modify the effects of ULT on developing T2DM risk. Further large studies need to be conducted to examine the interaction between co-morbidities and reduction of serum uric acid levels in developing T2DM risk.

We found a significant effect of ULT modification by sex, with the higher magnitude of the association among women than men. The mechanisms underlying the association of UA with the development of T2DM are not completely understood. Several potential pathophysiological mechanisms have been proposed, including inflammation, sex hormones, medication modification, and genetic effects. Ongoing low-grade inflammation in gout patients may promote the diabetogenic process. The differences in the baseline serum level between men and women and the difference in uric acid metabolism may explain the higher risk of diabetes in female gout patients compared with in male gout patients[12]. Sex hormones play a role in this regard. It is proposed that increased renal clearance of urate related to estrogen in premenopausal women may account for the lower SUA levels observed in women than in men [26]. A conceivable mechanism underlying the association between hyperuricemia and the risk of T2DM may occur at the renal level[11]. For example, urate transporter genes *ABCG2*, *SLC2A9*, and *SLC22A12* modulate the relationship of renal urate homeostasis and gout[27] with T2DM. Although Mendelian randomization studies have shown that uric acid-associated loci are causally associated with gout incidence [28], no evidence of a causal association between uric acid-associated loci and T2DM is available for European and South Asian populations[23, 28, 29]. In addition, we found that only benzbromarone use and combination therapy were associated with a higher T2DM risk in women compared to men (Table 4).These findings support the evidence that impact of gout on T2DM risk was higher among women. [12]. However, no such difference was found for allopurinol use. Uricosuric drugs may enhance the renal excretion of oxypurinol, the active metabolite of allopurinol, thus reducing the efficacy of allopurinol[30, 31].

According to a 15-year follow-up study, hyperuricemia often precedes the development of hyperinsulinemia, impaired fasting glucose, and diabetes, particularly in young adults [32]. The American College of Rheumatology guidelines recommends that plasma urate should be maintained at a concentration less than 360 μmol/L (6 mg/dL) for all patients on ULT[33]. ULT is indicated for patients with recurrent gout attacks, chronic arthropathy, tophi, and gout with uric acid stones[15]. Although effective treatments exist that eliminate sodium urate crystals and “cure” the disease, the management of gout is often suboptimal[16]. Allopurinol and benzbromarone improve insulin resistance[8–10], and they may be beneficial in lowering the risk of type 2 diabetes. However, most doctors focus solely on managing acute attacks rather than long-term therapy, and only one-third to one-half of gout patients ever receive ULT[16, 34]; furthermore, adherence to ULT is often poor (10%–46%)[35, 36]. Studies have shown that only 30%–60% of patients are prescribed allopurinol 1 year after the initiation of therapy, and that the management of gout is frequently inappropriate[15, 37]. Studies have demonstrated that treatment provided to reach the serum urate target with dose escalation of ULT is effective for achieving the therapeutic target in most (82%–92%) gout patients [38–40]. The management of gout is poor in Taiwan, with only one in five affected people being treated with ULT[18], suggesting that uric acid-lowering therapies in gout patients may not be beneficial in lowering T2DM risk in Taiwan.

This study has some strengths and limitations. This study was performed using data from a large population-based database in Taiwan. Therefore, the findings are likely applicable to the general population. Because the definition of gout and T2DM is based on ICD-9-CM diagnoses, a certain level of misclassification of exposure is inevitable. The identification of gout patients was based on ICD codes without the examination of synovial fluid or tophus aspirate for monosodium urate crystals, which may have led to some misclassification bias. We used a strict definition of gout requiring three clinic visits with ULT, which was found to have a high positive predictive value for fulfilling the various classification criteria for gout in primary care [41]. Several important covariates that are also associated with the risk of type 2 diabetes and gout(eg blood pressure, body mass index, use of diuretics, diet, consumption of alcohol, glucose levels, cholesterol, triglycerides, creatinine), could not be taken into account because of a lack of data. There is, thus, a possible risk for underestimation of some comorbidities, in particular hypertension, hyperlipidemia, obesity renal disease, and alcohol-related diseases, when ICD codes are used for definitions. In addition, we could not ascertain whether the patients had continued exposure to ULT beyond the index prescription date; thus, an immortal time bias may occur[42]. Nevertheless, we excluded immortal time by defining the start of follow-up for the treated group as the start of treatment for all subjects who received the urate-lowering drugs under investigation. Thus, patients were enrolled in the cohort at the time of their first prescription. Because of the lack of uric acid level at baseline, a detection bias may have occurred because persons with gout having higher uric acid values may more often visit doctors compared with those who are well-controlled. This could increase the likelihood of being diagnosed with T2DM. Individuals with T2DM have lower numbers of missing values for possible confounders such as hypertension, hyperlipidemia, obesity and renal disease. This may have led to residual confounding. Finally, although all included studies adjusted for a wide range of potential confounders for risk of developing T2DM, we cannot definitively exclude possible residual confounding effects because serum biochemistry, lifestyle, nutrition, and physical activity, are not routinely recorded in the NHIRD.

In conclusion, this study used data from a population-based database to determine a substantial association between ULT and a higher T2DM risk, particularly in gout patients younger than 50 years and those with prolonged ULT and a high dose of ULT. Despite the availability of effective ULT, only 22.93% of gout patients are prescribed urate-lowering treatment in Taiwan, which remained unchanged between 2005 and 2010[18], possibly contributing to elevated urate doses and increased gout flares with major adverse consequences such as T2DM. These findings support appropriate recognition and management of the risk factor of T2DM by using ULT for hyperuricemia in gout patients.

## Supporting information

S1 Table Allopurinol and benzbromarone use in gout patients. (DOCX)

S2 Table Association between allopurinol or benzbromarone use by annual exposure day and developing T2DM risk. (DOCX)
