# Supplementary material for: Associations between urate-lowering therapy and the risk of type 2 diabetes mellitus
Source: PLoS One. 2019 Jan 7;14(1):e0210085. doi: 10.1371/journal.pone.0210085 (PMC6322774; doi:10.1371/journal.pone.0210085)
Supplement: S1 Table — (DOCX) [file pone.0210085.s001.docx]

**S1 Table. Allopurinol and benzbromarone use in gout patients**.

|  |  | Inhibiting uric acid production  M04AA01 (Allopurinol) | | |  |  | Increasing uric acid excretion  M04AB03(Benzbromarone) | | |  |
| --- | --- | --- | --- | --- | --- | --- | --- | --- | --- | --- |
|  | Total | Type 2 diabetes |  | Non-type 2 diabetes | P | Total | Type 2 diabetes |  | Non-type 2 diabetes | P |
| Males , n | 14589 | 1668 |  | 12921 |  | 21042 | 2255 |  | 18787 |  |
| Follow-up duration, median(IQR), years | 7.7(4.5-10.7) | 5.3(3.1-8.1) |  | 8.2(4.8-11) | <0.0001 | 7.6(4.5-10.4) | 5.2(3-7.7) |  | 7.9(4.9-10.6) | <0.0001 |
| Total clinic visits, median(IQR), frequencies | 4(2-12) | 5(2-14) |  | 4(2-12) | 0.0033 | 5(2-14) | 7(3-16) |  | 5(2-14) | <0.0001 |
| Total drug use, median (IQR), days | 56(16-201) | 60(16-222) |  | 56(16-198) | 0.0496 | 112(42-308) | 120(42-346) |  | 109(42-303) | 0.0226 |
| Total drug tablets, median (IQR), quantities | 90(28-315) | 112(30-357) |  | 89(28-308) | 0.0024 | 126(55-353) | 152(56-406) |  | 122(54-345) | 0.0001 |
| Total dosage, median (IQR), mg | 9300(3000-33600) | 11600(3000-36700) |  | 9100(3000-33300) | 0.0127 | 8000(3000-21450) | 9150(3000-24500) |  | 7750(3000-21000) | 0.0014 |
| Total dosage(mg) /Follow-up duration(years×365 days) | 4.5(1.4-15.6) | 7.4(2.1-22.2) |  | 4.2(1.3-14.7) | <0.0001 | 3.7(1.4-10) | 5.6(2-14.7) |  | 3.5(1.3-9.5) | <0.0001 |
| Total drug use(day)/Follow-up duration(years) | 9.4(2.7-36.1) | 14(3.8-53.6) |  | 8.8(2.6-34.3) | <0.0001 | 19.1(7.1-52.2) | 29.4(9.9-73.8) |  | 18.2(6.8-49.4) | <0.0001 |
| Females , n | 2610 | 530 |  | 2080 |  | 4212 | 858 |  | 3354 |  |
| Follow-up duration, median(IQR), years | 7(3.9-10) | 4.6(2.6-6.9) |  | 7.6(4.6-10.6) | <0.0001 | 6.9(3.9-9.5) | 4.5(2.6-6.8) |  | 7.5(4.6-10.1) | <0.0001 |
| Total clinic visits, median(IQR), frequencies | 4(2-11) | 4(2-13) |  | 4(2-10) | 0.3079 | 5(2-12) | 5(2-12) |  | 5(2-12) | 0.29 |
| Total drug use, median (IQR), days | 58(16-200) | 56(18-210) |  | 58(15-196) | 0.7552 | 104(37-275) | 100.5(37-266) |  | 104(38-280) | 0.5321 |
| Total drug tablets, median (IQR), quantities | 85.5(28-278) | 90(28-327) |  | 84.3(28-269) | 0.2655 | 120(45-300) | 120(44-300) |  | 119.5(46-301) | 0.9503 |
| Total dosage, median (IQR), mg | 9000(2800-29100) | 9050(3000-33000) |  | 9000(2800-28000) | 0.4123 | 7200(3000-18575) | 7025(2950-17200) |  | 7250(3000-19200) | 0.4994 |
| Total dosage(mg) /Follow-up duration(years×365 days) | 4.5(1.3-16.6) | 7.2(1.9-27.5) |  | 4.1(1.2-14.2) | <0.0001 | 3.9(1.3-10.3) | 5.2(2-13.8) |  | 3.6(1.2-9.7) | <0.0001 |
| Total drug use(day)/Follow-up duration(years) | 10.7(2.8-42) | 15.4(4-69.9) |  | 9.6(2.6-36.9) | <0.0001 | 20.1(7.2-55.5) | 27(10.4-73) |  | 18.5(6.5-50.6) | <0.0001 |
| Combined group, n | 17199 | 2198 |  | 15001 |  | 25254 | 3113 |  | 22141 |  |
| Follow-up duration, median(IQR), years | 7.6(4.4-10.6) | 5.2(3-7.8) |  | 8.1(4.8-10.9) | <0.0001 | 7.4(4.4-10.3) | 5(2.9-7.4) |  | 7.8(4.8-10.6) | <0.0001 |
| Total clinic visits, median(IQR), frequencies | 4(2-12) | 5(2-14) |  | 4(2-12) | 0.0050 | 5(2-13) | 6(3-15) |  | 5(2-13) | <0.0001 |
| Total drug use, median (IQR), days | 56(16-201) | 60(17-220) |  | 56(16-198) | 0.0629 | 110(41-301) | 118(40-328) |  | 108(41-300) | 0.1575 |
| Total drug tablets, median (IQR), quantities | 90(28-308) | 105(30-346) |  | 88(28-300) | 0.0032 | 123(52-344) | 144(54-372) |  | 120(52-338) | 0.0045 |
| Total dosage, median (IQR), mg | 9300(3000-33000) | 11150(3000-35700) |  | 9100(3000-32600) | 0.0187 | 7750(3000-21000) | 8650(3000-22500) |  | 7700(3000-21000) | 0.0466 |
| Total dosage(mg) /Follow-up duration(years×365 days) | 4.5(1.4-15.8) | 7.3(2.1-23) |  | 4.2(1.3-14.6) | <0.0001 | 3.8(1.4-10) | 5.5(2-14.5) |  | 3.5(1.3-9.5) | <0.0001 |
| Total drug use(day)/Follow-up duration(years) | 9.6(2.7-36.9) | 14.3(3.9-54.9) |  | 8.9(2.6-34.6) | <0.0001 | 19.2(7.1-52.7) | 28.5(10.1-73.5) |  | 18.3(6.8-49.5) | <0.0001 |

IQR: interquartile range.

Continuous variables were analyzed using a *t* test for comparisons between type 2 diabetes and non-type 2 diabetes among gout patients.
