# Supplementary material for: Associations between urate-lowering therapy and the risk of type 2 diabetes mellitus
Source: PLoS One. 2019 Jan 7;14(1):e0210085. doi: 10.1371/journal.pone.0210085 (PMC6322774; doi:10.1371/journal.pone.0210085)
Supplement: S2 Table — (DOCX) [file pone.0210085.s002.docx]

**S2 Table. Association between allopurinol or benzbromarone use by annual exposure day and developing T2DM risk.**

|  | Type 2 diabetes  events, n (%) | Total people, n | Adjusted RR  (95% CI) | P value |
| --- | --- | --- | --- | --- |
| Allopurinol (M04AA01) |  |  |  |  |
| Annual exposure day |  |  |  |  |
| Non use | 6334 (10.64) | 59530 | 1.00 |  |
| Use, day |  |  |  |  |
| >0-2.5 | 89 (8.75) | 1017 | 0.89 (0.73-1.10) | 0.2959 |
| >2.5 to 8.8 | 131 (12.76) | 1027 | 1.24 (1.04-1.47) | 0.0161 |
| >8.8 to 37.0 | 150 (14.62) | 1026 | 1.27 (1.08-1.49) | 0.0039 |
| **>37.0** | **181 (17.69)** | **1023** | **1.23 (1.06-1.42)** | **0.0074** |
| Increasing uric acid excretion | 1501 (11.94) | 12566 | 1.09 (1.03-1.15) | 0.0036 |
| Combination therapy | 1647 (12.57) | 13106 | 1.03 (0.98-1.09) | 0.2750 |
| Benzbromarone (M04AB03) |  |  |  |  |
| Annual exposure day |  |  |  |  |
| Non use | 6334 (10.64) | 59530 | 1.00 |  |
| Use, day |  |  |  |  |
| >0 to 6.6 | 256 (8.35) | 3065 | 0.84 (0.75-0.96) | 0.0083 |
| >6.6 to 17.6 | 311 (10.06) | 3091 | 0.96 (0.85-1.07) | 0.4322 |
| >17.6 to 49.1 | 388 (12.56) | 3089 | 1.13 (1.02-1.25) | 0.0182 |
| **>49.1** | **527 (17.12)** | **3078** | **1.38 (1.26-1.51)** | **<0.0001** |
| Allopurinol | 551 (13.46) | 4093 | 1.17 (1.07-1.28) | 0.0004 |
| Probenecid or Sulfinpyrazone | 19 (7.82) | 243 | 0.76 (0.48-1.19) | 0.2236 |
| Combination therapy | 1647 (12.57) | 13106 | 1.03 (0.98-1.09) | 0.2607 |
| Sensitivity analysis***** |  |  |  |  |
| Allopurinol (M04AA01) |  |  |  |  |
| Annual exposure day |  |  |  |  |
| Non use | 6334 (10.64) | 59530 | 1.00 |  |
| Use, day |  |  |  |  |
| >0-2.5 | 386 (9.44) | 4089 | 0.93 (0.84-1.03) | 0.1461 |
| >2.5 to 8.8 | 486 (11.45) | 4243 | 1.08 (0.98-1.18) | 0.1142 |
| >8.8 to 37.0 | 628 (13.72) | 4576 | 1.13 (1.04-1.22) | 0.0051 |
| **>37.0** | **698 (16.27)** | **4291** | **1.09 (1.01-1.18)** | **0.0314** |
| Other | 1501 (11.94) | 12566 | 1.09 (1.03-1.15) | 0.0034 |
| Benzbromarone (M04AB03) |  |  |  |  |
| Annual exposure day |  |  |  |  |
| Non use | 6334 (10.64) | 59530 | 1.00 |  |
| Use, day |  |  |  |  |
| >0 to 6.6 | 521 (8.78) | 5933 | 0.84 (0.77-0.92) | <0.0001 |
| >6.6 to 17.6 | 648 (10.66) | 6078 | 0.98 (0.90-1.06) | 0.5539 |
| >17.6 to 49.1 | 855 (13.00) | 6576 | 1.12 (1.04-1.20) | 0.0026 |
| **>49.1** | **1089 (16.33)** | **6667** | **1.25 (1.17-1.33)** | **<0.0001** |
| Other | 586 (12.99) | 4511 | 1.14 (1.05-1.24) | 0.0029 |

Drugs used for increasing uric acid excretion were probenecid (M04AB01), sulfinpyrazone (M04AB02), and benzbromarone(M04AB03)

Combination therapy was allopurinol and drugs used for increasing uric acid excretion

Adjusted RR was calculated and adjusted for age group, sex, region, and comorbidities by using a Cox proportional hazards regression model.

Annual exposure day of allopurinol or benzbromarone: the annual exposure day allopurinol or benzbromarone divided by the total follow-up years (by the first treat gout date until the index date of type 2 diabetes or to the study end).

*****Sensitivity analysis 1 was calculated as all allopurinol or benzbromarone use in gout patients (versus controls).
